# Penta­kis­(μ_3_-*N*,2-di­oxido­benzene-1-car­box­imid­ato)di-μ_2_-formato-penta­kis­(1*H*-imidazole)­methanolpenta­manganese(III)man­gan­ese(II)–methanol–water (1/3.36/0.65)

**DOI:** 10.1107/S1600536812047228

**Published:** 2012-11-24

**Authors:** Benjamin R. Tigyer, Matthias Zeller, Curtis M. Zaleski

**Affiliations:** a Department of Chemistry, Shippensburg University, 1871 Old Main Dr., Shippensburg, PA 17257, USA; b Department of Chemistry, Youngstown State University, 1 University Plaza, Youngstown, OH 44555, USA

## Abstract

The title compound, [Mn_6_(C_7_H_4_NO_3_)_5_(CHO_2_)_2_(C_3_H_4_N_2_)_5_(CH_3_OH)]·3.36CH_3_OH·0.65H_2_O, or Mn(II)(O_2_CH)_2_[15-MC_Mn(III)N(shi)_-5](Im)_5_(MeOH)·3.36MeOH·0.65H_2_O (where MC is metallacrown, shi^3−^ is salicyl­hydroximate, Im is imidazole and MeOH is methanol), contains five Mn^III^ ions as members of the metallacrown ring and an Mn^II^ atom bound in the central cavity. The central Mn^II^ atom is seven-coordinate with a geometry best described as between face-capped trigonal–prismatic and face-capped octa­hedral. Three Mn^III^ ions of the metallacrown ring are six-coordinate with distorted octa­hedral geometries. Of these six-coordinate Mn^III^ ions, two have mirror-plane configurations, while the other has a Δ absolute stereoconfiguration. The remaining two Mn^III^ ions have a coordination number of five with a distorted square-pyramidal geometry. The five imidazole ligands are bound to five different Mn^III^ ions. Disorder is observed for one of the coordinating imidazole ligands, as the imidazole ligand is disordered over two alternative mutually exclusive positions in a ratio of 0.672 (9) to 0.328 (9). The inter­stitial voids between the main mol­ecules that constitute the structure are mostly filled with methanol mol­ecules that form hydrogen-bonded chains. Some of the sites of the non-coordinated methanol mol­ecules are not fully occupied, with the remainder of the volume either empty or taken up by ill-defined close to amorphous content. One site was refined as being taken up by either two or one methanol mol­ecules, with an occupancy ratio of 0.628 (5) to 0.343 (5). This disorder might thus be correlated with the disorder of the imidazole ring (an N—H⋯O hydrogen bond between the major moieties of the imidazole and the methanol mol­ecules is observed). On the other side of the disordered imidazole ring the chain of partially occupied methanol mol­ecules originates that extends *via* O—H⋯O hydrogen bonds to the metal-coordinated methanol mol­ecule. The three partially occupied methanol mol­ecules were refined to be disordered with two water mol­ecules to take two residual electron density peaks into account (the exact nature of these weak residual electron density peaks cannot be deduced from the X-ray diffraction data alone, the assignment as water is tentative). The occupancy rate for the methanol mol­ecules refined to 0.480 (7). The occupancy rate of the two water mol­ecules refined to 0.34 (1) and 0.31 (2) for each site.

## Related literature

For a general review of metallacrowns, see: Mezei *et al.* (2007 ▶). For related Mn(II)[15-MC_Mn(III)N(shi)_-5)] structures and related synthetic procedures, see: Kessissoglou *et al.* (1994 ▶); Dendrinou-Samara *et al.* (2001 ▶, 2002 ▶, 2005 ▶); Emerich *et al.* (2010 ▶); Tigyer *et al.* (2011 ▶). For an explanation on how to calculate τ, see: Addison *et al.* (1984 ▶). For an explanation on how to calculate the *s/h* ratio, see: Stiefel & Brown (1972 ▶).
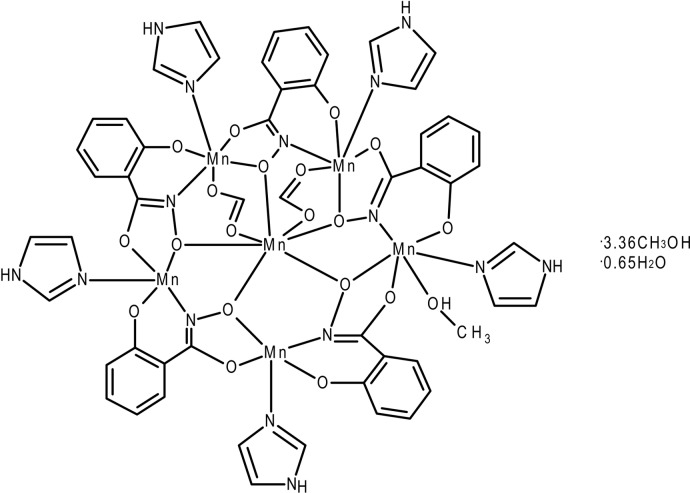


## Experimental

### 

#### Crystal data

[Mn_6_(C_7_H_4_NO_3_)_5_(CHO_2_)_2_(C_3_H_4_N_2_)_5_(CH_4_O)]·3.36CH_4_O·0.65H_2_O
*M* *_r_* = 1662.43
Monoclinic, 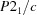

*a* = 13.2053 (12) Å
*b* = 24.621 (2) Å
*c* = 21.491 (2) Å
β = 101.861 (1)°
*V* = 6838.0 (11) Å^3^
*Z* = 4
Mo *K*α radiation
μ = 1.16 mm^−1^
*T* = 100 K
0.45 × 0.38 × 0.25 mm

#### Data collection

Bruker SMART APEX CCD diffractometer
Absorption correction: multi-scan (*TWINABS*; Sheldrick, 2009 ▶) *T*_min_ = 0.619, *T*_max_ = 0.746
87486 measured reflections
17626 independent reflections
14038 reflections with *I* > 2σ(*I*)
*R*_int_ = 0.044

#### Refinement

*R*[*F* ^2^ > 2σ(*F* ^2^)] = 0.049
*wR*(*F* ^2^) = 0.140
*S* = 1.05
17626 reflections
1006 parameters
26 restraints
H atoms treated by a mixture of independent and constrained refinement
Δρ_max_ = 2.17 e Å^−3^
Δρ_min_ = −0.59 e Å^−3^

### 

Data collection: *APEX2* (Bruker, 2012 ▶); cell refinement: *SAINT* (Bruker, 2012 ▶) and *CELL_NOW* (Sheldrick, 2008*b* ▶); data reduction: *SAINT*; program(s) used to solve structure: *SHELXS97* (Sheldrick, 2008*a* ▶); program(s) used to refine structure: *SHELXL2012* (Sheldrick, 2012 ▶), *SHELXLE* (Hübschle *et al.*, 2011 ▶); molecular graphics: *Mercury* (Macrae *et al.*, 2006 ▶) and *ORTEP-3 for Windows* (Farrugia, 2012) ▶; software used to prepare material for publication: *publCIF* (Westrip, 2010 ▶).

## Supplementary Material

Crystal structure: contains datablock(s) I, global. DOI: 10.1107/S1600536812047228/pk2457sup1.cif

Structure factors: contains datablock(s) I. DOI: 10.1107/S1600536812047228/pk2457Isup2.hkl

Additional supplementary materials:  crystallographic information; 3D view; checkCIF report

## Figures and Tables

**Table 1:** Hydrogen-bond geometry (Å, °)

*D*—H⋯*A*	*D*—H	H⋯*A*	*D*⋯*A*	*D*—H⋯*A*
C44—H44⋯O11	0.95	2.47	3.413 (4)	173
C47—H47⋯N14	0.95	2.68	3.599 (5)	162
C51—H51*A*⋯O18	0.98	2.50	3.360 (4)	147
N7—H7⋯O25*B*	0.88	1.88	2.658 (9)	147
N9—H9⋯O17^i^	0.88	2.07	2.898 (3)	156
N11—H11*A*⋯O14^ii^	0.88	2.00	2.869 (3)	168
N13—H13*A*⋯O2^iii^	0.88	1.99	2.827 (4)	159
N15—H15⋯O8^iv^	0.88	1.96	2.800 (4)	159
N7*B*—H7*B*⋯O21	0.88	2.11	2.933 (15)	155
O20—H20*A*⋯O22^v^	0.85 (2)	1.85 (2)	2.681 (5)	168 (5)
O20—H20*A*⋯O22*B* ^v^	0.85 (2)	1.96 (4)	2.75 (3)	155 (4)
O22—H22*A*⋯O24	0.84	1.97	2.746 (7)	154
O24—H24*A*⋯O21	0.84	2.03	2.808 (6)	154
O25*B*—H25*A*⋯O23*B*	0.84	1.91	2.601 (9)	138
O22*B*—H22*C*⋯O21*B* ^vi^	0.84	2.48	3.29 (4)	160
O23—H23⋯O12^vii^	0.84	2.19	2.828 (9)	133
O23*B*—H23*B*⋯O12^vii^	0.84	2.08	2.897 (6)	165

Symmetry codes: (i) 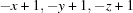; (ii) 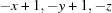; (iii) 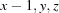; (iv) 
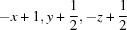
; (v) 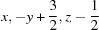; (vi) 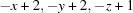; (vii) 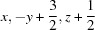.

**Fig. 1.**
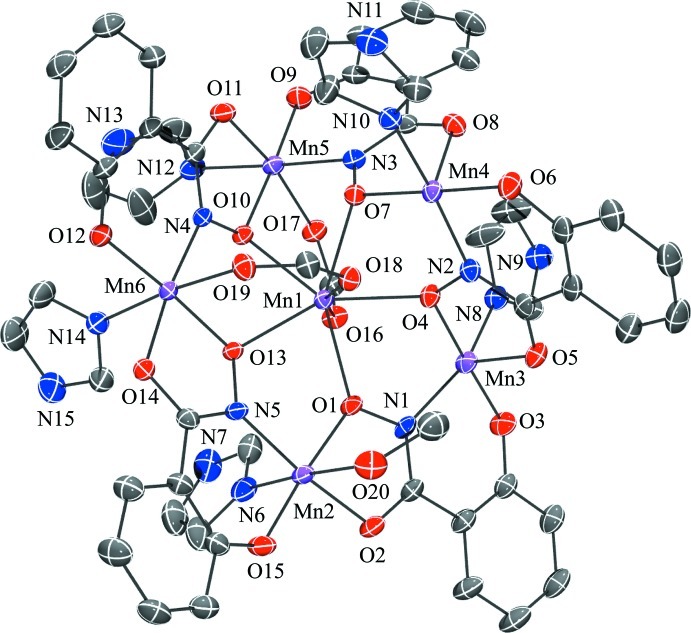
Single-crystal X-ray structure of Mn(II)(O2CH)2[15-MCMn(III)N(shi)-5](Im)5(MeOH).3.36MeOH.0.65H2O (1). The thermal ellipsoid plot of 1 is at a 50% probability level. Hydrogen atoms and the lattice solvent molecules have been omitted for clarity. The disordered atoms of the imidazole attached to Mn2 are only shown at the higher occupancy positions. Color scheme for all figures: purple - Mn(II) and Mn(III), red - oxygen, blue - nitrogen, and gray - carbon.

**Fig. 2.**
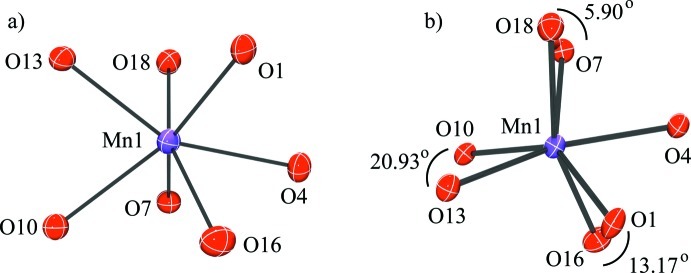
Side (a) and top (b) views of the first coordination sphere about Mn1 (2+ oxidation state) of 1. The thermal ellipsoid plots are at a 50% probability level.

**Fig. 3.**
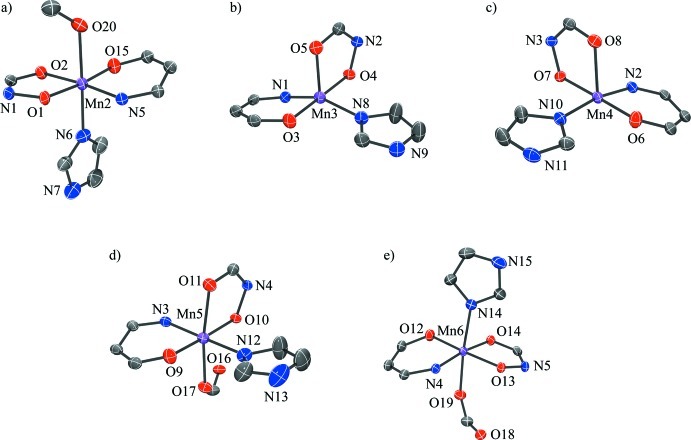
First coordination sphere about each Mn(III) ion of 1. a) Mn2 with planar configuration b) Mn3 with distorted square pyramidal geometry c) Mn4 with distorted square pyramidal geometry d) Mn5 with Δ configuration and e) Mn6 with planar configuration. The thermal ellipsoid plots are at a 50% probability level. Hydrogen atoms have been omitted for clarity. The disordered atoms of the imidazole attached to Mn2 are only shown at the higher occupancy positions.

## supplementary crystallographic information

### Comment

Metallacrowns (MCs), in particular 15-MC-5 complexes, have a variety of potential applications, spanning single-molecule magnetism, ion selectivity, and antibacterial activity (Mezei *et al.*, 2007). The family of manganese-based 15-MC-5 complexes, which consists of a 15 membered ring with a -[Mn(III)—N—O]_5_– repeat unit and a central Mn(II) ion, have shown better antibacterial activity than simple manganese-herbicide complexes (Dendrinou-Samara *et al.*, 2002, 2005). The first few Mn-based 15-MC-5 complexes were made with pyridine molecules bound to the structure (Kessissoglou *et al.*, 1994; Dendrinou-Samara *et al.*, 2001, 2002, 2005); however, it has recently been shown that imidazole can also be used to produce a Mn-based 15-MC-5 complex (Emerich *et al.*, 2010; Tigyer *et al.* 2011).

Herein we report the synthesis, IR data, and the single-crystal X-ray structure of the title compound, [Mn_6_(C_7_H_4_NO_3_)_5_(C_3_N_2_H_4_)_5_(CH_4_O)(CHO_2_)_2_]^.^3.36CH_3_OH^.^0.65H_2_O, 1, abbreviated as Mn(II)(O_2_CH)_2_[15-MC_Mn(III)N(shi)_-5](Im)_5_(MeOH)^.^3.36MeOH^.^0.65H_2_O (where MC is metallacrown, shi^3-^ is salicylhydroximate, Im is imidazole, and MeOH is methanol). Compound 1 is a non-planar molecule, which is typical of Mn(II)[15-MC_Mn(III)_-5] structures (Fig. 1–3; Farrugia, 1997). The structure consists of a –[Mn(III)—N—O]_5_– repeat unit around the MC ring, and the MC binds a Mn(II) in the central cavity (Fig. 1). The positive charge of the five Mn(III) ions and the one Mn(II) ion are counterbalanced by the five shi^3-^ ligands and two formate anions.

Mn1 is located in the central cavity and is seven-coordinate with a geometry best described as between face-capped trigonal prismatic and face-capped octahedral (Fig. 2). The geometry is significantly distorted when compared to the ideal parameters of either geometry. One parameter that can be used to distinguish these geometries is the azimuthal angle (Φ). In a trigonal prism the ideal angle between the atoms on opposite triangular faces is Φ = 0°, while for an octahedron the ideal value is Φ = 60°. To calculate the Φ angle, the centroids of opposite triangular faces made by the donor oxygen atoms (O1, O13, and O18; O7, O10, and O16) were defined using the program *Mercury* (Macrae *et al.*, 2006), and then twist angles between atoms on opposite faces through the centroids were calculated. [A similar method was used to calculate the Φ angle in a related 15-MC-5 structure (Tigyer *et al.*, 2011).] The estimated Φ angles of 5.90°, 13.17°, and 20.93° indicate that the geometry approaches that of a face-capped trigonal prism though the angle of 20.93° is significantly large (Fig. 2). Another parameter that can be used to distinguish the two geometries is the *s/h* ratio (Stiefel and Brown, 1972). In an ideal octahedron the *s/h* ratio is 1.22, while in an ideal trigonal prism the *s/h* ratio is 1.00. Defining the distance between the centroids as *h* and defining the distances between atoms within a triangular face as *s*, the estimated average *s/h* ratio for 1 is 1.15± 0.13. When considered together, the Φ angle and the *s/h* ratio indicate that the geometry about Mn1 is severely distorted from both face-capped trigonal prismatic and face-capped octahedral geometry. The assignment of a 2+ oxidation state for Mn1 is supported by an average bond distance of 2.24 Å.

The ring Mn2 - Mn6 (Fig. 3) are assigned a 3+ oxidation state, which is supported by the average bond distances. The average Mn-N/O bond distances for Mn2, Mn3, Mn4, Mn5, and Mn6 are 2.03 Å, 1.96 Å, 1.98 Å, 2.03 Å, and 2.03 Å, respectively. Mn2, Mn5, and Mn6 are six-coordinate and possess a Jahn-Teller axis, which is typical for a high spin *d*^4^ cation further supporting a 3+ oxidation state (Fig. 3a-c). The average bond distances of Mn3 and Mn4 are shorter than those of the other ring Mn ions; however, Mn3 and Mn4 are only five coordinate (Fig. 3 d-e). In previous Mn(II)[15-MC_Mn(III)_-5] structures, the coordination spheres of the Mn ions had been completed by forming bonds to the oxygen atoms of the carboxylate anions (Kessissoglou *et al.*, 1994; Dendrinou-Samara *et al.*, 2001, 2002, 2005; Emerich *et al.*, 2010; and Tigyer *et al.*, 2011); however, in 1 these bonds do not exist. The geometry about Mn2, Mn5, and Mn6 is best described as a distorted octahedron (Fig. 3a-c). The coordination about these Mn(III) ions can also be described by their configurations. Mn2 and Mn6 adopt a planar (P) configuration, where two chelate rings of different shi^3-^ ligands are located *trans* to each other. Mn5 has a propeller configuration with Δ absolute stereochemistry. The geometry about Mn3 and Mn4 is best described as distorted square pyramidal (Fig. d-e). To evaluate the geometry about Mn3 and Mn4 the τ parameter was calculated for both Mn(III) ions (Addison *et al.*, 1984). For an ideal square pyramidal geometry τ = 0, while for an ideal trigonal bipyramidal geometry τ = 1. For Mn3 and Mn4, τ equals 0.38 and 0.15, respectively. In addition, Mn2, Mn3, Mn4, Mn5, and Mn6 bind imidazole ligands, which are directed to the periphery of the molecule. The imidazole attached to Mn2 is disordered over two alternative mutually exclusive positions in a ratio of 0.672 (9) to 0.328 (9).

### Experimental

Manganese(II) chloride tetrahydrate (99%), salicylhydroxamic acid (H_3_shi, 99%), and sodium formate (98%) were purchased from Alfa Aesar. Imidazole (ReagentPlus, 99%) was purchased from Sigma-Aldrich. Sodium methoxide was purchased from Matheson Coleman and Bell. Methanol (HPLC grade) was purchased from Pharmco-AAPer. All reagents were used as received and without further purification.

Manganese(II) chloride tetrahydrate (3.0 mmol) was dissolved in 30 ml of methanol resulting in a light pink solution. Sodium methoxide (7.5 mmol) and H_3_shi (2.5 mmol) were mixed in 20 ml of methanol, which resulted in a cloudy white liquid. This mixture was then added to the manganese(II) chloride solution. Initially the solution turned a yellow color, but after stirring for 1 h the solution become a dark brown-black. After 1 h of stirring, separate solutions of sodium formate (3.0 mmol in 20 ml of methanol) and imidazole (10 mmol in 30 ml of methanol) were added to the dark brown-black solution. No color change was observed. This final solution (100 ml total volume) was left for slow evaporation of the solvent at room temperature. Dark brown-black crystals suitable for X-ray diffraction analysis were collected after 1 day. The percent yield was 2.5% based on manganese(II) chloride tetrahydrate.

Elemental analysis for the dried material C_56.36_H_60.77_Mn_6_N_15_O_24.02_ [FW = 1662.28 g/mol] found % (calculated); C 39.58 (40.72); H 3.19 (3.69); N 12.74 (12.64).

### Refinement

Crystals of the compound were heavily intergrown. A mostly single piece was extracted from a larger cluster, but due to the dark color and fragility of the material no completely single fragment of sufficient size could be obtained. The crystal chosen for data collection thus consisted of several fragments, two of which were dominant. The orientation matrices for the two major components were identified using the program *CELL_NOW* (Sheldrick, 2008*b*), with the two components being related by no obvious twin law [18.4 degrees about reciprocal axis (-0.374 - 0.889 1.000) or real axis (-0.745 - 0.736 1.000)]. The two components were integrated using *SAINT* (Bruker, 2012), resulting in a total of 148319 reflections. 61126 reflections (16635 unique) involved component 1 only (mean I/σ = 10.2), 60494 reflections (16623 unique) involved component 2 only (mean I/σ = 3.1), 26606 reflections (15709 unique) involved both components (mean I/σ = 8.9). The transformation matrix identified by the integration program was found to be (0.91781 -0.10681 -0.12487, 0.46932 0.96639 0.14355, 0.28545 -0.10318 1.01409.

The data were corrected for absorption using *TWINABS*, and the structure was solved and refined using direct methods using only the non-overlapping reflections of component 1 with a resolution better than 0.7 Å. Overlapping reflections were ignored. The *R*_int_ value given is for all reflections and is based on agreement between observed single intensities of component 1 before the cutoff at 0.7 Å [*TWINABS* (Sheldrick, 2009)].

Disorder is observed for one of the coordinated imidazole ligands and for the solvate methanol and water molecules. The imidazole ligand is disordered over two alternative, mutually exclusive positions in a ratio of 0.672 (9) to 0.328 (9). The geometries of the two moieties were restrained to be similar and ADPs of partially overlapping atoms of the two moieties were constrained to be identical. The interstitial voids between the main molecules that constitute the structure are mostly filled with methanol molecules that form hydrogen bonded chains. Some of the sites of the non-coordinated methanol molecules are not fully occupied, with the remainder of the volume either empty or ill-defined. One site was refined as being taken up by either two or one methanol molecules, with an occupancy ratio of 0.628 (5) to 0.343 (5). This disorder might thus be correlated with the disorder of the imidazole ring (an N—H···O hydrogen bond between the major moieties of the imidazole and the methanol molecules is observed). On the other side of the disordered imidazole ring a chain of partially occupied methanol molecules originates that extends *via* O—H···O hydrogen bonds to the metal coordinated methanol molecule. The three partially occupied methanol molecules were refined to be disordered with two water molecules to take two residual electron density peaks into account (the exact nature of these weak residual electron density peaks cannot be deduced from the X-ray diffraction data alone, the assignment as water is tentative). The occupancy of the methanol molecules refined to 0.480 (7). The occupancy of the two water molecules refined to 0.34 (1) and 0.31 (2). The ADPs of the water molecules were restrained to be approximately isotropic. Acidic H atoms were set based on hydrogen bonding considerations and were restrained or constrained.

### Crystal data

**Table:**

[Mn_6_(C_7_H_4_NO_3_)_5_(CHO_2_)_2_(C_3_H_4_N_2_)_5_(CH_4_O)]·3.36CH_4_O·0.65H_2_O	*F*(000) = 3384.9
*M**_r_* = 1662.43	*D*_x_ = 1.615 Mg m^−^^3^
Monoclinic, *P*2_1_/*c*	Mo *K*α radiation, λ = 0.71073 Å
*a* = 13.2053 (12) Å	Cell parameters from 2337 reflections
*b* = 24.621 (2) Å	θ = 2.3–24.6°
*c* = 21.491 (2) Å	µ = 1.16 mm^−^^1^
β = 101.861 (1)°	*T* = 100 K
*V* = 6838.0 (11) Å^3^	Fragment, black
*Z* = 4	0.45 × 0.38 × 0.25 mm

### Data collection

**Table:**

Bruker SMART APEX CCD diffractometer	17626 independent reflections
Radiation source: sealed tube	14038 reflections with *I* > 2σ(*I*)
Graphite monochromator	*R*_int_ = 0.044
φ and ω scans	θ_max_ = 28.8°, θ_min_ = 1.6°
Absorption correction: multi-scan (*TWINABS*; Sheldrick, 2009)	*h* = −17→17
*T*_min_ = 0.619, *T*_max_ = 0.746	*k* = −33→33
87486 measured reflections	*l* = −29→29

### Refinement

**Table:**

Refinement on *F*^2^	Primary atom site location: structure-invariant direct methods
Least-squares matrix: full	Secondary atom site location: difference Fourier map
*R*[*F*^2^ > 2σ(*F*^2^)] = 0.049	Hydrogen site location: difference Fourier map
*wR*(*F*^2^) = 0.140	H atoms treated by a mixture of independent and constrained refinement
*S* = 1.05	*w* = 1/[σ^2^(*F*_o_^2^) + (0.0719*P*)^2^ + 12.0988*P*] where *P* = (*F*_o_^2^ + 2*F*_c_^2^)/3
17626 reflections	(Δ/σ)_max_ = 0.001
1006 parameters	Δρ_max_ = 2.17 e Å^−^^3^
26 restraints	Δρ_min_ = −0.59 e Å^−^^3^

### Special details

**Table:**

Experimental. FT–IR bands (KBr pellet, cm^-1^): 1597(*s*), 1569(*s*), 1498(*s*), 1436(*m*), 1387(*m*), 1317(*m*), 1257(*m*), 1244(*m*), 1145(*w*), 1100(*w*), 1065(*m*), 1021(*w*), 927(*m*), 861(*m*), 756(*m*), 679(*m*), 648(*m*), 611(*m*), 576(*w*) and 471(*w*).
Geometry. All e.s.d.'s (except the e.s.d. in the dihedral angle between two l.s. planes) are estimated using the full covariance matrix. The cell e.s.d.'s are taken into account individually in the estimation of e.s.d.'s in distances, angles and torsion angles; correlations between e.s.d.'s in cell parameters are only used when they are defined by crystal symmetry. An approximate (isotropic) treatment of cell e.s.d.'s is used for estimating e.s.d.'s involving l.s. planes.

### Fractional atomic coordinates and isotropic or equivalent isotropic displacement parameters (Å^2^)

**Table:**

	*x*	*y*	*z*	*U*_iso_*/*U*_eq_	Occ. (<1)
C1	0.8891 (2)	0.63065 (12)	0.35278 (13)	0.0229 (6)	
C2	0.9638 (2)	0.62374 (13)	0.41336 (14)	0.0267 (6)	
C3	1.0536 (2)	0.65587 (16)	0.42408 (16)	0.0350 (7)	
H3	1.0637	0.6814	0.3927	0.042*	
C4	1.1280 (3)	0.65093 (18)	0.47991 (17)	0.0416 (9)	
H4	1.1884	0.6729	0.4869	0.050*	
C5	1.1126 (3)	0.61337 (16)	0.52527 (16)	0.0379 (8)	
H5	1.1640	0.6090	0.5630	0.045*	
C6	1.0240 (3)	0.58227 (15)	0.51651 (15)	0.0334 (7)	
H6	1.0146	0.5573	0.5486	0.040*	
C7	0.9473 (2)	0.58698 (13)	0.46063 (14)	0.0266 (6)	
C8	0.7939 (2)	0.45047 (12)	0.30564 (13)	0.0227 (6)	
C10	0.9389 (3)	0.38975 (15)	0.29828 (16)	0.0327 (7)	
H10	0.9786	0.4095	0.3328	0.039*	
C11	0.9849 (3)	0.34801 (15)	0.27189 (18)	0.0380 (8)	
H11	1.0555	0.3393	0.2878	0.046*	
C12	0.9274 (3)	0.31891 (14)	0.22206 (17)	0.0352 (7)	
H12	0.9589	0.2904	0.2031	0.042*	
C13	0.8238 (3)	0.33118 (13)	0.19956 (15)	0.0309 (7)	
H13	0.7845	0.3104	0.1658	0.037*	
C14	0.7766 (2)	0.37371 (12)	0.22588 (14)	0.0257 (6)	
C15	0.4263 (2)	0.43909 (12)	0.28414 (13)	0.0222 (5)	
C16	0.3500 (2)	0.42548 (12)	0.32361 (13)	0.0248 (6)	
C17	0.3641 (3)	0.37713 (13)	0.35851 (15)	0.0305 (7)	
H17	0.4221	0.3548	0.3567	0.037*	
C18	0.2950 (3)	0.36120 (14)	0.39581 (16)	0.0374 (8)	
H18	0.3060	0.3286	0.4198	0.045*	
C19	0.2100 (3)	0.39356 (15)	0.39743 (17)	0.0378 (8)	
H19	0.1617	0.3827	0.4223	0.045*	
C20	0.1943 (3)	0.44126 (14)	0.36356 (16)	0.0328 (7)	
H20	0.1347	0.4626	0.3647	0.039*	
C21	0.2655 (2)	0.45898 (13)	0.32717 (14)	0.0265 (6)	
C22	0.3082 (2)	0.56798 (11)	0.12815 (14)	0.0223 (5)	
O21	0.8131 (3)	0.93158 (15)	0.33972 (19)	0.0344 (10)	0.628 (5)
H21	0.7774	0.9601	0.3368	0.052*	0.628 (5)
C52	0.9110 (5)	0.9431 (3)	0.3220 (3)	0.0410 (15)	0.628 (5)
H52A	0.8982	0.9597	0.2797	0.061*	0.628 (5)
H52B	0.9497	0.9092	0.3214	0.061*	0.628 (5)
H52C	0.9512	0.9681	0.3530	0.061*	0.628 (5)
O24	0.8778 (4)	0.8676 (2)	0.4488 (2)	0.0647 (17)	0.628 (5)
H24A	0.8674	0.8944	0.4242	0.097*	0.628 (5)
C55	0.9450 (5)	0.8320 (3)	0.4285 (3)	0.0526 (18)	0.628 (5)
H55A	0.9092	0.8136	0.3897	0.079*	0.628 (5)
H55B	0.9690	0.8050	0.4617	0.079*	0.628 (5)
H55C	1.0044	0.8522	0.4195	0.079*	0.628 (5)
O21B	0.9130 (9)	0.9711 (8)	0.3556 (8)	0.119 (7)	0.343 (14)
H21A	0.9383	0.9677	0.3227	0.179*	0.343 (14)
H21B	0.8999	0.9389	0.3658	0.179*	0.343 (14)
O22B	1.011 (2)	0.9039 (13)	0.6068 (17)	0.202 (16)	0.312 (18)
H22B	0.9595	0.9097	0.5771	0.303*	0.312 (18)
H22C	1.0267	0.9338	0.6254	0.303*	0.312 (18)
C23	0.2719 (2)	0.56834 (12)	0.05786 (14)	0.0245 (6)	
C24	0.1996 (3)	0.52789 (13)	0.03172 (16)	0.0317 (7)	
H24	0.1796	0.5013	0.0589	0.038*	
O23B	0.3617 (5)	0.7457 (3)	0.4810 (3)	0.0413 (16)	0.480 (7)
H23B	0.3687	0.7736	0.5040	0.062*	0.480 (7)
C54B	0.4202 (8)	0.7015 (4)	0.5154 (5)	0.050 (2)	0.480 (7)
H54D	0.4912	0.7135	0.5325	0.075*	0.480 (7)
H54E	0.4212	0.6708	0.4866	0.075*	0.480 (7)
H54F	0.3877	0.6903	0.5505	0.075*	0.480 (7)
O25B	0.4553 (6)	0.7510 (3)	0.3859 (4)	0.065 (2)	0.480 (7)
H25A	0.4473	0.7365	0.4199	0.097*	0.480 (7)
C56	0.3895 (7)	0.7252 (3)	0.3334 (4)	0.048 (2)	0.480 (7)
H56A	0.3455	0.6986	0.3492	0.071*	0.480 (7)
H56B	0.4318	0.7066	0.3074	0.071*	0.480 (7)
H56C	0.3460	0.7525	0.3076	0.071*	0.480 (7)
C54	0.4111 (13)	0.7231 (5)	0.4600 (9)	0.116 (6)	0.520 (7)
H54A	0.4824	0.7270	0.4543	0.175*	0.520 (7)
H54B	0.3760	0.6949	0.4313	0.175*	0.520 (7)
H54C	0.4114	0.7128	0.5041	0.175*	0.520 (7)
O23	0.3626 (16)	0.7688 (4)	0.4472 (5)	0.169 (8)	0.520 (7)
H23	0.3408	0.7793	0.4791	0.254*	0.520 (7)
C25	0.1573 (3)	0.52635 (15)	−0.03309 (17)	0.0373 (8)	
H25	0.1104	0.4983	−0.0503	0.045*	
C26	0.1837 (3)	0.56553 (15)	−0.07169 (16)	0.0359 (7)	
H26	0.1535	0.5652	−0.1158	0.043*	
C27	0.2536 (3)	0.60559 (15)	−0.04741 (16)	0.0343 (7)	
H27	0.2708	0.6325	−0.0751	0.041*	
C28	0.3000 (2)	0.60729 (13)	0.01746 (14)	0.0263 (6)	
C29	0.6614 (2)	0.68927 (11)	0.11018 (13)	0.0208 (5)	
C30	0.7473 (2)	0.71630 (12)	0.08805 (14)	0.0239 (6)	
C31	0.7312 (3)	0.73208 (13)	0.02375 (15)	0.0306 (7)	
H31	0.6674	0.7237	−0.0040	0.037*	
C32	0.8064 (3)	0.75950 (15)	0.00036 (16)	0.0367 (8)	
H32	0.7943	0.7699	−0.0431	0.044*	
C33	0.8997 (3)	0.77191 (14)	0.04061 (17)	0.0343 (7)	
H33	0.9518	0.7906	0.0246	0.041*	
C34	0.9172 (2)	0.75726 (13)	0.10377 (16)	0.0301 (6)	
H34	0.9815	0.7661	0.1308	0.036*	
C35	0.8418 (2)	0.72948 (12)	0.12913 (14)	0.0248 (6)	
N6	0.7275 (2)	0.73654 (11)	0.28385 (13)	0.0314 (6)	0.672 (9)
C36	0.6610 (9)	0.7253 (3)	0.3220 (5)	0.0346 (15)	0.672 (9)
H36	0.6432	0.6900	0.3339	0.042*	0.672 (9)
N7	0.6237 (5)	0.7725 (3)	0.3405 (2)	0.0440 (14)	0.672 (9)
H7	0.5787	0.7760	0.3653	0.053*	0.672 (9)
C37	0.6696 (7)	0.8142 (3)	0.3131 (4)	0.051 (2)	0.672 (9)
H37	0.6568	0.8519	0.3165	0.061*	0.672 (9)
C38	0.736 (2)	0.7913 (5)	0.2808 (13)	0.0445 (19)	0.672 (9)
H38	0.7812	0.8104	0.2594	0.053*	0.672 (9)
N6B	0.7275 (2)	0.73654 (11)	0.28385 (13)	0.0314 (6)	0.328 (9)
C36B	0.750 (5)	0.7894 (9)	0.272 (3)	0.0445 (19)	0.328 (9)
H36B	0.7867	0.7997	0.2406	0.053*	0.328 (9)
N7B	0.7145 (13)	0.8253 (6)	0.3100 (8)	0.051 (2)	0.328 (9)
H7B	0.7253	0.8606	0.3126	0.061*	0.328 (9)
C37B	0.6587 (13)	0.7946 (6)	0.3431 (7)	0.0440 (14)	0.328 (9)
H37B	0.6219	0.8080	0.3735	0.053*	0.328 (9)
C38B	0.664 (2)	0.7411 (7)	0.3257 (12)	0.0346 (15)	0.328 (9)
H38B	0.6283	0.7119	0.3407	0.042*	0.328 (9)
C39	0.7138 (3)	0.48765 (14)	0.50398 (15)	0.0328 (7)	
H39	0.7737	0.5027	0.5305	0.039*	
C40	0.5695 (3)	0.4435 (2)	0.47325 (18)	0.0531 (12)	
H40	0.5095	0.4223	0.4733	0.064*	
C41	0.5979 (3)	0.4661 (2)	0.42223 (17)	0.0500 (11)	
H41	0.5607	0.4631	0.3796	0.060*	
C42	0.5134 (2)	0.37829 (13)	0.09220 (14)	0.0274 (6)	
H42	0.5728	0.3556	0.0993	0.033*	
C43	0.3672 (3)	0.41483 (15)	0.04707 (16)	0.0378 (8)	
H43	0.3053	0.4228	0.0172	0.045*	
C44	0.4023 (3)	0.44043 (14)	0.10313 (16)	0.0342 (7)	
H44	0.3687	0.4694	0.1199	0.041*	
C45	0.1423 (3)	0.61267 (16)	0.2780 (2)	0.0441 (9)	
H45	0.1161	0.5827	0.2976	0.053*	
C46	0.1524 (4)	0.69199 (19)	0.2355 (2)	0.0591 (12)	
H46	0.1361	0.7277	0.2197	0.071*	
C47	0.2395 (3)	0.66375 (17)	0.2345 (3)	0.0563 (12)	
H47	0.2964	0.6764	0.2178	0.068*	
C48	0.4669 (3)	0.76009 (13)	0.17921 (16)	0.0313 (7)	
H48	0.5376	0.7547	0.1986	0.038*	
C49	0.3140 (3)	0.79703 (16)	0.1512 (2)	0.0504 (10)	
H49	0.2574	0.8216	0.1470	0.060*	
C50	0.3134 (3)	0.74740 (15)	0.1248 (2)	0.0431 (9)	
H50	0.2554	0.7310	0.0979	0.052*	
C51	0.8901 (3)	0.54796 (15)	0.20866 (19)	0.0395 (8)	
H51A	0.8270	0.5284	0.1891	0.059*	
H51B	0.9013	0.5448	0.2550	0.059*	
H51C	0.9493	0.5323	0.1940	0.059*	
O22	0.9303 (4)	0.8901 (2)	0.5765 (2)	0.0440 (12)	0.628 (5)
H22A	0.9027	0.8919	0.5377	0.066*	0.628 (5)
C53	1.0359 (6)	0.8776 (4)	0.5832 (4)	0.056 (2)	0.628 (5)
H53A	1.0468	0.8390	0.5932	0.084*	0.628 (5)
H53B	1.0764	0.8994	0.6177	0.084*	0.628 (5)
H53C	1.0580	0.8858	0.5434	0.084*	0.628 (5)
C77	0.6028 (2)	0.53445 (12)	0.10639 (14)	0.0242 (6)	
H77	0.6232	0.5079	0.0793	0.029*	
C79	0.5060 (2)	0.59516 (13)	0.37450 (14)	0.0277 (6)	
H79	0.5272	0.6060	0.4176	0.033*	
N1	0.80399 (18)	0.60208 (10)	0.34002 (11)	0.0217 (5)	
N2	0.69836 (19)	0.46630 (10)	0.28295 (11)	0.0213 (5)	
N3	0.42489 (18)	0.48870 (10)	0.26086 (11)	0.0207 (5)	
N4	0.38732 (17)	0.59966 (9)	0.15326 (10)	0.0185 (4)	
N5	0.67536 (17)	0.66868 (10)	0.16729 (11)	0.0200 (4)	
N8	0.6888 (2)	0.49410 (11)	0.44156 (12)	0.0270 (5)	
N9	0.6439 (2)	0.45729 (12)	0.52472 (12)	0.0331 (6)	
H9	0.6456	0.4478	0.5644	0.040*	
C9	0.8358 (2)	0.40383 (12)	0.27589 (14)	0.0246 (6)	
N10	0.49506 (19)	0.41718 (10)	0.13155 (11)	0.0224 (5)	
N11	0.4364 (2)	0.37593 (12)	0.04158 (12)	0.0312 (6)	
H11A	0.4316	0.3528	0.0098	0.037*	
N12	0.2325 (2)	0.61383 (11)	0.26148 (13)	0.0288 (5)	
N13	0.0931 (2)	0.65925 (14)	0.2635 (2)	0.0513 (9)	
H13A	0.0318	0.6675	0.2709	0.062*	
N14	0.4091 (2)	0.72421 (10)	0.14255 (13)	0.0265 (5)	
N15	0.4130 (3)	0.80504 (12)	0.18542 (15)	0.0411 (7)	
H15	0.4365	0.8343	0.2073	0.049*	
O1	0.74153 (15)	0.61515 (8)	0.28045 (9)	0.0213 (4)	
O2	0.90941 (15)	0.66591 (9)	0.31135 (10)	0.0253 (4)	
O3	0.86226 (17)	0.55681 (10)	0.45636 (10)	0.0300 (5)	
O4	0.67127 (15)	0.51340 (8)	0.31239 (9)	0.0212 (4)	
O5	0.85109 (16)	0.47592 (9)	0.35204 (10)	0.0268 (4)	
O6	0.67632 (17)	0.38320 (9)	0.20187 (11)	0.0299 (5)	
O7	0.49745 (15)	0.49752 (8)	0.22320 (9)	0.0201 (4)	
O8	0.49295 (16)	0.40416 (8)	0.27295 (10)	0.0249 (4)	
O9	0.24548 (16)	0.50657 (9)	0.29765 (11)	0.0290 (5)	
O10	0.41738 (14)	0.59720 (8)	0.22028 (9)	0.0187 (4)	
O11	0.26424 (16)	0.53760 (9)	0.16274 (10)	0.0263 (4)	
O12	0.36874 (16)	0.64722 (9)	0.03760 (10)	0.0267 (4)	
O13	0.58379 (14)	0.64878 (8)	0.18359 (9)	0.0179 (4)	
O14	0.57102 (15)	0.68663 (8)	0.07192 (9)	0.0211 (4)	
O15	0.86388 (16)	0.71894 (9)	0.19178 (10)	0.0290 (5)	
O16	0.57211 (16)	0.60067 (9)	0.34087 (9)	0.0256 (4)	
O17	0.41548 (16)	0.57726 (9)	0.35890 (10)	0.0266 (4)	
O18	0.63380 (15)	0.52696 (8)	0.16619 (9)	0.0220 (4)	
O19	0.54934 (17)	0.57240 (8)	0.07982 (10)	0.0256 (4)	
O20	0.87965 (19)	0.60394 (10)	0.19089 (12)	0.0349 (5)	
H20A	0.902 (3)	0.6023 (19)	0.1569 (14)	0.052*	
Mn1	0.58751 (3)	0.57528 (2)	0.24447 (2)	0.01690 (9)	
Mn2	0.79807 (3)	0.67029 (2)	0.23644 (2)	0.02070 (10)	
Mn3	0.76730 (3)	0.53645 (2)	0.38493 (2)	0.02250 (10)	
Mn4	0.58914 (3)	0.43996 (2)	0.21334 (2)	0.01998 (10)	
Mn5	0.33219 (3)	0.55126 (2)	0.26207 (2)	0.02037 (10)	
Mn6	0.47204 (3)	0.64620 (2)	0.11103 (2)	0.01828 (9)	

### Atomic displacement parameters (Å^2^)

**Table:**

	*U*^11^	*U*^22^	*U*^33^	*U*^12^	*U*^13^	*U*^23^
C1	0.0175 (13)	0.0322 (15)	0.0182 (13)	0.0018 (11)	0.0021 (10)	−0.0010 (11)
C2	0.0208 (14)	0.0361 (16)	0.0209 (14)	0.0006 (12)	−0.0012 (11)	−0.0040 (12)
C3	0.0219 (15)	0.052 (2)	0.0289 (16)	−0.0081 (14)	−0.0004 (12)	0.0002 (15)
C4	0.0224 (16)	0.064 (2)	0.0341 (18)	−0.0058 (15)	−0.0038 (14)	−0.0013 (17)
C5	0.0257 (16)	0.055 (2)	0.0269 (16)	0.0046 (15)	−0.0091 (13)	−0.0030 (15)
C6	0.0318 (17)	0.0432 (19)	0.0213 (15)	0.0053 (14)	−0.0040 (12)	−0.0016 (13)
C7	0.0237 (14)	0.0331 (16)	0.0202 (13)	0.0027 (12)	−0.0016 (11)	−0.0031 (11)
C8	0.0254 (14)	0.0254 (14)	0.0170 (12)	0.0012 (11)	0.0034 (11)	0.0068 (10)
C10	0.0270 (16)	0.0402 (18)	0.0302 (16)	0.0050 (13)	0.0046 (13)	0.0060 (13)
C11	0.0273 (16)	0.044 (2)	0.044 (2)	0.0131 (14)	0.0094 (14)	0.0084 (16)
C12	0.0368 (18)	0.0318 (16)	0.0402 (18)	0.0115 (14)	0.0153 (15)	0.0069 (14)
C13	0.0390 (18)	0.0278 (15)	0.0267 (15)	0.0068 (13)	0.0089 (13)	0.0045 (12)
C14	0.0279 (15)	0.0251 (14)	0.0243 (14)	0.0050 (11)	0.0056 (12)	0.0084 (11)
C15	0.0231 (14)	0.0238 (13)	0.0179 (13)	−0.0052 (11)	0.0002 (10)	−0.0011 (10)
C16	0.0289 (15)	0.0268 (14)	0.0189 (13)	−0.0088 (11)	0.0053 (11)	−0.0019 (11)
C17	0.0435 (18)	0.0258 (15)	0.0229 (14)	−0.0076 (13)	0.0083 (13)	−0.0017 (12)
C18	0.056 (2)	0.0292 (16)	0.0303 (16)	−0.0118 (15)	0.0173 (16)	0.0022 (13)
C19	0.048 (2)	0.0385 (18)	0.0325 (17)	−0.0156 (16)	0.0212 (16)	−0.0041 (14)
C20	0.0333 (17)	0.0371 (17)	0.0304 (16)	−0.0118 (14)	0.0123 (13)	−0.0075 (13)
C21	0.0283 (15)	0.0285 (15)	0.0228 (14)	−0.0107 (12)	0.0055 (12)	−0.0037 (11)
C22	0.0199 (13)	0.0215 (13)	0.0235 (14)	0.0005 (10)	−0.0002 (11)	−0.0012 (10)
O21	0.041 (2)	0.0267 (19)	0.038 (2)	0.0068 (15)	0.0150 (17)	0.0048 (15)
C52	0.028 (3)	0.052 (4)	0.038 (3)	0.012 (3)	−0.004 (2)	0.000 (3)
O24	0.069 (3)	0.091 (4)	0.037 (3)	0.039 (3)	0.020 (2)	0.023 (3)
C55	0.045 (4)	0.067 (5)	0.046 (4)	0.003 (3)	0.010 (3)	0.014 (3)
O21B	0.046 (7)	0.190 (16)	0.113 (12)	0.040 (9)	−0.004 (7)	0.046 (10)
O22B	0.21 (2)	0.19 (2)	0.23 (2)	−0.015 (15)	0.097 (17)	0.093 (15)
C23	0.0221 (14)	0.0261 (14)	0.0217 (14)	−0.0014 (11)	−0.0039 (11)	−0.0025 (11)
C24	0.0312 (16)	0.0289 (16)	0.0309 (16)	−0.0066 (13)	−0.0027 (13)	0.0006 (13)
O23B	0.046 (3)	0.039 (3)	0.040 (3)	−0.001 (3)	0.011 (3)	−0.013 (3)
C54B	0.069 (6)	0.038 (4)	0.046 (5)	0.009 (4)	0.019 (4)	−0.009 (4)
O25B	0.076 (5)	0.058 (4)	0.069 (5)	−0.033 (4)	0.034 (4)	−0.021 (3)
C56	0.055 (5)	0.037 (4)	0.052 (5)	0.009 (4)	0.013 (4)	0.000 (4)
C54	0.151 (14)	0.059 (8)	0.161 (16)	−0.009 (8)	0.085 (13)	−0.047 (9)
O23	0.39 (2)	0.051 (5)	0.050 (5)	−0.010 (8)	−0.004 (8)	−0.007 (4)
C25	0.0347 (18)	0.0390 (18)	0.0322 (17)	−0.0076 (14)	−0.0071 (14)	−0.0085 (14)
C26	0.0322 (17)	0.047 (2)	0.0237 (15)	−0.0032 (14)	−0.0062 (13)	−0.0023 (14)
C27	0.0291 (16)	0.046 (2)	0.0249 (15)	−0.0046 (14)	−0.0018 (13)	0.0058 (14)
C28	0.0211 (14)	0.0294 (15)	0.0251 (14)	−0.0020 (11)	−0.0030 (11)	−0.0013 (11)
C29	0.0248 (14)	0.0171 (12)	0.0199 (13)	−0.0029 (10)	0.0029 (11)	−0.0028 (10)
C30	0.0260 (14)	0.0222 (13)	0.0248 (14)	−0.0044 (11)	0.0083 (11)	0.0005 (11)
C31	0.0357 (17)	0.0317 (16)	0.0240 (15)	−0.0088 (13)	0.0053 (13)	−0.0007 (12)
C32	0.0438 (19)	0.0414 (19)	0.0261 (15)	−0.0117 (15)	0.0098 (14)	0.0028 (14)
C33	0.0352 (17)	0.0315 (16)	0.0399 (18)	−0.0098 (13)	0.0164 (15)	0.0018 (14)
C34	0.0278 (15)	0.0276 (15)	0.0351 (17)	−0.0063 (12)	0.0066 (13)	−0.0025 (13)
C35	0.0263 (14)	0.0223 (13)	0.0260 (14)	−0.0034 (11)	0.0063 (11)	0.0014 (11)
N6	0.0257 (13)	0.0321 (14)	0.0331 (14)	0.0002 (11)	−0.0015 (11)	−0.0084 (11)
C36	0.041 (2)	0.032 (5)	0.027 (2)	0.011 (4)	−0.0014 (18)	−0.012 (4)
N7	0.054 (4)	0.037 (3)	0.037 (2)	0.011 (2)	−0.001 (2)	−0.014 (2)
C37	0.065 (6)	0.032 (4)	0.050 (3)	0.011 (4)	0.001 (5)	−0.002 (3)
C38	0.058 (9)	0.027 (2)	0.042 (7)	0.007 (3)	−0.003 (3)	−0.008 (2)
N6B	0.0257 (13)	0.0321 (14)	0.0331 (14)	0.0002 (11)	−0.0015 (11)	−0.0084 (11)
C36B	0.058 (9)	0.027 (2)	0.042 (7)	0.007 (3)	−0.003 (3)	−0.008 (2)
N7B	0.065 (6)	0.032 (4)	0.050 (3)	0.011 (4)	0.001 (5)	−0.002 (3)
C37B	0.054 (4)	0.037 (3)	0.037 (2)	0.011 (2)	−0.001 (2)	−0.014 (2)
C38B	0.041 (2)	0.032 (5)	0.027 (2)	0.011 (4)	−0.0014 (18)	−0.012 (4)
C39	0.0400 (18)	0.0379 (18)	0.0190 (14)	−0.0064 (14)	0.0025 (13)	0.0005 (12)
C40	0.052 (2)	0.080 (3)	0.0252 (17)	−0.032 (2)	0.0042 (16)	0.0047 (18)
C41	0.039 (2)	0.085 (3)	0.0214 (16)	−0.029 (2)	−0.0032 (14)	0.0083 (18)
C42	0.0304 (15)	0.0290 (15)	0.0234 (14)	−0.0019 (12)	0.0064 (12)	−0.0039 (11)
C43	0.0412 (19)	0.0419 (19)	0.0253 (16)	0.0053 (15)	−0.0050 (14)	−0.0024 (14)
C44	0.0316 (17)	0.0359 (17)	0.0310 (17)	0.0066 (13)	−0.0035 (13)	−0.0044 (13)
C45	0.0267 (17)	0.042 (2)	0.067 (3)	−0.0017 (15)	0.0192 (17)	−0.0076 (18)
C46	0.056 (3)	0.050 (2)	0.077 (3)	0.028 (2)	0.026 (2)	0.011 (2)
C47	0.045 (2)	0.044 (2)	0.092 (4)	0.0194 (18)	0.041 (2)	0.020 (2)
C48	0.0319 (16)	0.0258 (15)	0.0340 (17)	0.0005 (12)	0.0015 (13)	−0.0023 (12)
C49	0.046 (2)	0.0341 (19)	0.066 (3)	0.0153 (17)	0.002 (2)	−0.0056 (18)
C50	0.0294 (17)	0.0308 (17)	0.062 (2)	0.0076 (14)	−0.0075 (16)	−0.0078 (16)
C51	0.0338 (18)	0.0398 (19)	0.045 (2)	−0.0001 (15)	0.0084 (15)	−0.0060 (16)
O22	0.046 (3)	0.057 (3)	0.034 (2)	0.007 (2)	0.0199 (19)	0.0097 (19)
C53	0.041 (4)	0.063 (5)	0.076 (5)	−0.019 (3)	0.038 (4)	−0.020 (4)
C77	0.0301 (15)	0.0234 (14)	0.0204 (13)	0.0035 (11)	0.0082 (11)	−0.0001 (11)
C79	0.0269 (15)	0.0352 (16)	0.0217 (14)	−0.0004 (12)	0.0064 (12)	−0.0051 (12)
N1	0.0171 (11)	0.0316 (13)	0.0139 (10)	0.0005 (9)	−0.0023 (8)	0.0007 (9)
N2	0.0238 (12)	0.0218 (11)	0.0174 (11)	0.0017 (9)	0.0025 (9)	0.0013 (9)
N3	0.0203 (11)	0.0243 (12)	0.0183 (11)	−0.0018 (9)	0.0057 (9)	−0.0004 (9)
N4	0.0182 (11)	0.0195 (11)	0.0157 (10)	−0.0008 (8)	−0.0016 (8)	0.0005 (8)
N5	0.0154 (10)	0.0229 (11)	0.0211 (11)	−0.0050 (8)	0.0028 (9)	0.0005 (9)
N8	0.0295 (13)	0.0316 (13)	0.0184 (11)	−0.0020 (10)	0.0017 (10)	0.0015 (10)
N9	0.0380 (15)	0.0424 (16)	0.0180 (12)	−0.0051 (12)	0.0034 (11)	0.0035 (11)
C9	0.0261 (14)	0.0266 (14)	0.0215 (13)	0.0057 (11)	0.0057 (11)	0.0077 (11)
N10	0.0239 (12)	0.0223 (11)	0.0194 (11)	−0.0016 (9)	0.0007 (9)	0.0001 (9)
N11	0.0385 (15)	0.0346 (14)	0.0198 (12)	−0.0057 (12)	0.0046 (11)	−0.0054 (10)
N12	0.0207 (12)	0.0332 (14)	0.0337 (14)	−0.0001 (10)	0.0084 (10)	−0.0022 (11)
N13	0.0242 (15)	0.050 (2)	0.083 (3)	0.0061 (13)	0.0168 (16)	−0.0130 (18)
N14	0.0245 (12)	0.0215 (12)	0.0317 (13)	0.0007 (9)	0.0019 (10)	−0.0014 (10)
N15	0.0501 (19)	0.0269 (14)	0.0430 (17)	0.0047 (13)	0.0014 (14)	−0.0084 (12)
O1	0.0176 (9)	0.0290 (10)	0.0148 (9)	−0.0039 (8)	−0.0023 (7)	0.0028 (7)
O2	0.0170 (9)	0.0375 (12)	0.0198 (10)	−0.0042 (8)	0.0003 (8)	0.0019 (8)
O3	0.0309 (11)	0.0394 (12)	0.0158 (9)	−0.0061 (9)	−0.0042 (8)	0.0013 (9)
O4	0.0225 (10)	0.0238 (10)	0.0157 (9)	0.0028 (8)	0.0001 (7)	−0.0005 (7)
O5	0.0219 (10)	0.0329 (11)	0.0227 (10)	0.0036 (8)	−0.0024 (8)	0.0013 (8)
O6	0.0284 (11)	0.0270 (11)	0.0314 (11)	0.0069 (9)	−0.0009 (9)	−0.0071 (9)
O7	0.0190 (9)	0.0207 (9)	0.0211 (9)	−0.0005 (7)	0.0052 (7)	0.0014 (7)
O8	0.0298 (11)	0.0219 (10)	0.0230 (10)	−0.0024 (8)	0.0054 (8)	0.0018 (8)
O9	0.0233 (10)	0.0305 (11)	0.0355 (12)	−0.0039 (9)	0.0114 (9)	0.0047 (9)
O10	0.0171 (9)	0.0226 (9)	0.0154 (9)	−0.0015 (7)	0.0014 (7)	−0.0006 (7)
O11	0.0244 (10)	0.0285 (11)	0.0240 (10)	−0.0084 (8)	0.0003 (8)	0.0005 (8)
O12	0.0249 (10)	0.0289 (11)	0.0220 (10)	−0.0051 (8)	−0.0050 (8)	0.0061 (8)
O13	0.0149 (9)	0.0218 (9)	0.0165 (9)	−0.0030 (7)	0.0022 (7)	0.0023 (7)
O14	0.0222 (10)	0.0215 (9)	0.0172 (9)	−0.0019 (7)	−0.0018 (7)	0.0002 (7)
O15	0.0242 (11)	0.0332 (12)	0.0280 (11)	−0.0114 (9)	0.0014 (9)	0.0019 (9)
O16	0.0266 (11)	0.0319 (11)	0.0191 (9)	−0.0026 (8)	0.0069 (8)	−0.0041 (8)
O17	0.0226 (10)	0.0353 (12)	0.0220 (10)	−0.0030 (9)	0.0047 (8)	0.0005 (9)
O18	0.0231 (10)	0.0244 (10)	0.0188 (9)	0.0015 (8)	0.0050 (8)	0.0019 (8)
O19	0.0325 (11)	0.0230 (10)	0.0206 (10)	0.0019 (8)	0.0038 (8)	0.0012 (8)
O20	0.0357 (13)	0.0401 (13)	0.0322 (12)	−0.0015 (10)	0.0147 (10)	−0.0035 (10)
Mn1	0.01652 (19)	0.01949 (19)	0.01425 (18)	−0.00034 (14)	0.00212 (14)	0.00079 (14)
Mn2	0.0167 (2)	0.0255 (2)	0.0185 (2)	−0.00469 (16)	0.00044 (15)	0.00038 (16)
Mn3	0.0227 (2)	0.0283 (2)	0.01402 (19)	−0.00149 (17)	−0.00181 (16)	0.00162 (16)
Mn4	0.0207 (2)	0.0205 (2)	0.0173 (2)	0.00172 (15)	0.00069 (16)	−0.00041 (15)
Mn5	0.0167 (2)	0.0234 (2)	0.0214 (2)	−0.00192 (15)	0.00466 (16)	−0.00014 (16)
Mn6	0.01722 (19)	0.01867 (19)	0.01691 (19)	−0.00124 (15)	−0.00124 (15)	0.00111 (15)

### Geometric parameters (Å, º)

**Table:**

C1—N1	1.307 (4)	N7—C37	1.383 (9)
C1—O2	1.310 (4)	N7—H7	0.8800
C1—C2	1.473 (4)	C37—C38	1.348 (13)
C2—C3	1.404 (4)	C37—H37	0.9500
C2—C7	1.411 (4)	C38—H38	0.9500
C3—C4	1.391 (5)	C36B—N7B	1.347 (19)
C3—H3	0.9500	C36B—H36B	0.9500
C4—C5	1.389 (5)	N7B—C37B	1.355 (15)
C4—H4	0.9500	N7B—H7B	0.8800
C5—C6	1.379 (5)	C37B—C38B	1.375 (16)
C5—H5	0.9500	C37B—H37B	0.9500
C6—C7	1.408 (4)	C38B—H38B	0.9500
C6—H6	0.9500	C39—N8	1.324 (4)
C7—O3	1.333 (4)	C39—N9	1.334 (4)
C8—O5	1.284 (4)	C39—H39	0.9500
C8—N2	1.315 (4)	C40—C41	1.349 (5)
C8—C9	1.476 (4)	C40—N9	1.363 (5)
C10—C11	1.374 (5)	C40—H40	0.9500
C10—C9	1.391 (4)	C41—N8	1.373 (4)
C10—H10	0.9500	C41—H41	0.9500
C11—C12	1.379 (5)	C42—N11	1.329 (4)
C11—H11	0.9500	C42—N10	1.332 (4)
C12—C13	1.387 (5)	C42—H42	0.9500
C12—H12	0.9500	C43—N11	1.346 (5)
C13—C14	1.396 (4)	C43—C44	1.354 (5)
C13—H13	0.9500	C43—H43	0.9500
C14—O6	1.340 (4)	C44—N10	1.376 (4)
C14—C9	1.404 (4)	C44—H44	0.9500
C15—O8	1.288 (4)	C45—N12	1.311 (4)
C15—N3	1.319 (4)	C45—N13	1.324 (5)
C15—C16	1.482 (4)	C45—H45	0.9500
C16—C17	1.399 (4)	C46—C47	1.348 (5)
C16—C21	1.403 (5)	C46—N13	1.348 (6)
C17—C18	1.390 (5)	C46—H46	0.9500
C17—H17	0.9500	C47—N12	1.370 (5)
C18—C19	1.383 (6)	C47—H47	0.9500
C18—H18	0.9500	C48—N14	1.318 (4)
C19—C20	1.375 (5)	C48—N15	1.337 (4)
C19—H19	0.9500	C48—H48	0.9500
C20—C21	1.410 (4)	C49—C50	1.347 (5)
C20—H20	0.9500	C49—N15	1.377 (5)
C21—O9	1.333 (4)	C49—H49	0.9500
C22—O11	1.275 (4)	C50—N14	1.367 (4)
C22—N4	1.327 (4)	C50—H50	0.9500
C22—C23	1.488 (4)	C51—O20	1.429 (5)
O21—C52	1.449 (8)	C51—H51A	0.9800
O21—H21	0.8400	C51—H51B	0.9800
C52—H52A	0.9800	C51—H51C	0.9800
C52—H52B	0.9800	O22—C53	1.405 (9)
C52—H52C	0.9800	O22—H22A	0.8400
O24—C55	1.380 (8)	C53—H53A	0.9800
O24—H24A	0.8400	C53—H53B	0.9800
C55—H55A	0.9800	C53—H53C	0.9800
C55—H55B	0.9800	C77—O19	1.238 (4)
C55—H55C	0.9800	C77—O18	1.279 (3)
O21B—H21A	0.8480	C77—H77	0.9500
O21B—H21B	0.8507	C79—O16	1.249 (4)
O22B—H22B	0.8389	C79—O17	1.254 (4)
O22B—H22C	0.8434	C79—H79	0.9500
C23—C28	1.394 (4)	N1—O1	1.410 (3)
C23—C24	1.414 (4)	N1—Mn3	1.992 (3)
C24—C25	1.391 (5)	N2—O4	1.402 (3)
C24—H24	0.9500	N2—Mn4	1.963 (2)
O23B—C54B	1.446 (11)	N3—O7	1.393 (3)
O23B—H23B	0.8400	N3—Mn5	1.971 (2)
C54B—H54D	0.9800	N4—O10	1.415 (3)
C54B—H54E	0.9800	N4—Mn6	1.951 (2)
C54B—H54F	0.9800	N5—O13	1.414 (3)
O25B—C56	1.424 (11)	N5—Mn2	1.961 (2)
O25B—H25A	0.8400	N8—Mn3	2.040 (3)
C56—H56A	0.9800	N9—H9	0.8800
C56—H56B	0.9800	N10—Mn4	2.012 (2)
C56—H56C	0.9800	N11—H11A	0.8800
C54—O23	1.295 (18)	N12—Mn5	2.025 (3)
C54—H54A	0.9800	N13—H13A	0.8800
C54—H54B	0.9800	N14—Mn6	2.252 (3)
C54—H54C	0.9800	N15—H15	0.8800
O23—H23	0.8400	O1—Mn2	1.894 (2)
C25—C26	1.363 (5)	O1—Mn1	2.2476 (19)
C25—H25	0.9500	O2—Mn2	1.947 (2)
C26—C27	1.379 (5)	O3—Mn3	1.841 (2)
C26—H26	0.9500	O4—Mn3	1.8837 (19)
C27—C28	1.403 (4)	O4—Mn1	2.2387 (19)
C27—H27	0.9500	O5—Mn3	2.066 (2)
C28—O12	1.348 (4)	O6—Mn4	1.859 (2)
C29—O14	1.304 (3)	O7—Mn4	1.904 (2)
C29—N5	1.305 (4)	O7—Mn1	2.250 (2)
C29—C30	1.476 (4)	O8—Mn4	2.168 (2)
C30—C31	1.409 (4)	O9—Mn5	1.862 (2)
C30—C35	1.411 (4)	O10—Mn5	1.9407 (19)
C31—C32	1.378 (5)	O10—Mn1	2.2647 (19)
C31—H31	0.9500	O11—Mn5	2.165 (2)
C32—C33	1.387 (5)	O12—Mn6	1.862 (2)
C32—H32	0.9500	O13—Mn6	1.9150 (19)
C33—C34	1.377 (5)	O13—Mn1	2.2276 (19)
C33—H33	0.9500	O14—Mn6	1.964 (2)
C34—C35	1.406 (4)	O15—Mn2	1.858 (2)
C34—H34	0.9500	O16—Mn1	2.213 (2)
C35—O15	1.343 (4)	O17—Mn5	2.238 (2)
N6—C36	1.347 (11)	O18—Mn1	2.245 (2)
N6—C38	1.355 (12)	O18—Mn4	2.492 (2)
N6—Mn2	2.227 (3)	O19—Mn6	2.253 (2)
C36—N7	1.355 (8)	O20—Mn2	2.285 (2)
C36—H36	0.9500	O20—H20A	0.847 (19)
			
N1—C1—O2	120.1 (2)	C50—C49—N15	106.1 (3)
N1—C1—C2	120.9 (3)	C50—C49—H49	126.9
O2—C1—C2	119.0 (3)	N15—C49—H49	126.9
C3—C2—C7	119.6 (3)	C49—C50—N14	109.7 (3)
C3—C2—C1	118.0 (3)	C49—C50—H50	125.2
C7—C2—C1	122.4 (3)	N14—C50—H50	125.2
C4—C3—C2	121.0 (3)	O20—C51—H51A	109.5
C4—C3—H3	119.5	O20—C51—H51B	109.5
C2—C3—H3	119.5	H51A—C51—H51B	109.5
C5—C4—C3	119.0 (3)	O20—C51—H51C	109.5
C5—C4—H4	120.5	H51A—C51—H51C	109.5
C3—C4—H4	120.5	H51B—C51—H51C	109.5
C6—C5—C4	121.1 (3)	C53—O22—H22A	109.5
C6—C5—H5	119.4	O22—C53—H53A	109.5
C4—C5—H5	119.4	O22—C53—H53B	109.5
C5—C6—C7	120.8 (3)	H53A—C53—H53B	109.5
C5—C6—H6	119.6	O22—C53—H53C	109.5
C7—C6—H6	119.6	H53A—C53—H53C	109.5
O3—C7—C6	117.5 (3)	H53B—C53—H53C	109.5
O3—C7—C2	124.1 (3)	O19—C77—O18	127.3 (3)
C6—C7—C2	118.4 (3)	O19—C77—H77	116.3
O5—C8—N2	120.6 (3)	O18—C77—H77	116.3
O5—C8—C9	120.2 (3)	O16—C79—O17	128.7 (3)
N2—C8—C9	119.1 (3)	O16—C79—H79	115.7
C11—C10—C9	121.8 (3)	O17—C79—H79	115.7
C11—C10—H10	119.1	C1—N1—O1	112.7 (2)
C9—C10—H10	119.1	C1—N1—Mn3	128.3 (2)
C10—C11—C12	119.3 (3)	O1—N1—Mn3	118.01 (17)
C10—C11—H11	120.3	C8—N2—O4	113.1 (2)
C12—C11—H11	120.3	C8—N2—Mn4	133.1 (2)
C11—C12—C13	120.2 (3)	O4—N2—Mn4	113.67 (16)
C11—C12—H12	119.9	C15—N3—O7	114.0 (2)
C13—C12—H12	119.9	C15—N3—Mn5	132.8 (2)
C12—C13—C14	120.8 (3)	O7—N3—Mn5	112.90 (16)
C12—C13—H13	119.6	C22—N4—O10	114.9 (2)
C14—C13—H13	119.6	C22—N4—Mn6	129.45 (19)
O6—C14—C13	117.6 (3)	O10—N4—Mn6	115.54 (15)
O6—C14—C9	123.6 (3)	C29—N5—O13	113.9 (2)
C13—C14—C9	118.8 (3)	C29—N5—Mn2	129.78 (19)
O8—C15—N3	120.3 (3)	O13—N5—Mn2	115.77 (16)
O8—C15—C16	121.8 (3)	C39—N8—C41	105.5 (3)
N3—C15—C16	117.9 (3)	C39—N8—Mn3	127.9 (2)
C17—C16—C21	119.4 (3)	C41—N8—Mn3	126.6 (2)
C17—C16—C15	117.9 (3)	C39—N9—C40	107.5 (3)
C21—C16—C15	122.7 (3)	C39—N9—H9	126.2
C18—C17—C16	121.2 (3)	C40—N9—H9	126.2
C18—C17—H17	119.4	C10—C9—C14	119.0 (3)
C16—C17—H17	119.4	C10—C9—C8	118.1 (3)
C19—C18—C17	119.0 (3)	C14—C9—C8	122.9 (3)
C19—C18—H18	120.5	C42—N10—C44	106.2 (3)
C17—C18—H18	120.5	C42—N10—Mn4	126.5 (2)
C20—C19—C18	120.9 (3)	C44—N10—Mn4	127.2 (2)
C20—C19—H19	119.6	C42—N11—C43	108.4 (3)
C18—C19—H19	119.6	C42—N11—H11A	125.8
C19—C20—C21	120.9 (3)	C43—N11—H11A	125.8
C19—C20—H20	119.6	C45—N12—C47	106.1 (3)
C21—C20—H20	119.6	C45—N12—Mn5	127.4 (3)
O9—C21—C16	124.9 (3)	C47—N12—Mn5	125.8 (2)
O9—C21—C20	116.6 (3)	C45—N13—C46	108.7 (3)
C16—C21—C20	118.5 (3)	C45—N13—H13A	125.6
O11—C22—N4	121.5 (3)	C46—N13—H13A	125.6
O11—C22—C23	120.4 (3)	C48—N14—C50	106.0 (3)
N4—C22—C23	118.1 (3)	C48—N14—Mn6	123.1 (2)
C52—O21—H21	109.5	C50—N14—Mn6	130.5 (2)
O21—C52—H52A	109.5	C48—N15—C49	107.1 (3)
O21—C52—H52B	109.5	C48—N15—H15	126.4
H52A—C52—H52B	109.5	C49—N15—H15	126.4
O21—C52—H52C	109.5	N1—O1—Mn2	113.57 (15)
H52A—C52—H52C	109.5	N1—O1—Mn1	122.13 (15)
H52B—C52—H52C	109.5	Mn2—O1—Mn1	124.27 (9)
C55—O24—H24A	109.5	C1—O2—Mn2	111.94 (17)
O24—C55—H55A	109.5	C7—O3—Mn3	128.96 (19)
O24—C55—H55B	109.5	N2—O4—Mn3	115.58 (15)
H55A—C55—H55B	109.5	N2—O4—Mn1	113.98 (14)
O24—C55—H55C	109.5	Mn3—O4—Mn1	119.53 (10)
H55A—C55—H55C	109.5	C8—O5—Mn3	110.32 (18)
H55B—C55—H55C	109.5	C14—O6—Mn4	132.1 (2)
H21A—O21B—H21B	104.8	N3—O7—Mn4	118.54 (16)
H22B—O22B—H22C	107.4	N3—O7—Mn1	114.37 (15)
C28—C23—C24	118.8 (3)	Mn4—O7—Mn1	109.53 (9)
C28—C23—C22	124.4 (3)	C15—O8—Mn4	110.17 (17)
C24—C23—C22	116.7 (3)	C21—O9—Mn5	129.6 (2)
C25—C24—C23	121.1 (3)	N4—O10—Mn5	115.35 (15)
C25—C24—H24	119.4	N4—O10—Mn1	107.54 (14)
C23—C24—H24	119.4	Mn5—O10—Mn1	113.39 (9)
C54B—O23B—H23B	109.5	C22—O11—Mn5	109.73 (17)
O23B—C54B—H54D	109.5	C28—O12—Mn6	126.18 (19)
O23B—C54B—H54E	109.5	N5—O13—Mn6	111.40 (14)
H54D—C54B—H54E	109.5	N5—O13—Mn1	120.69 (15)
O23B—C54B—H54F	109.5	Mn6—O13—Mn1	111.82 (9)
H54D—C54B—H54F	109.5	C29—O14—Mn6	111.01 (17)
H54E—C54B—H54F	109.5	C35—O15—Mn2	127.91 (19)
C56—O25B—H25A	109.5	C79—O16—Mn1	136.1 (2)
O25B—C56—H56A	109.5	C79—O17—Mn5	127.48 (19)
O25B—C56—H56B	109.5	C77—O18—Mn1	126.60 (18)
H56A—C56—H56B	109.5	C77—O18—Mn4	118.62 (18)
O25B—C56—H56C	109.5	Mn1—O18—Mn4	91.56 (7)
H56A—C56—H56C	109.5	C77—O19—Mn6	136.21 (19)
H56B—C56—H56C	109.5	C51—O20—Mn2	127.1 (2)
O23—C54—H54A	109.5	C51—O20—H20A	99 (3)
O23—C54—H54B	109.5	Mn2—O20—H20A	133 (3)
H54A—C54—H54B	109.5	O16—Mn1—O13	108.98 (8)
O23—C54—H54C	109.5	O16—Mn1—O4	73.15 (8)
H54A—C54—H54C	109.5	O13—Mn1—O4	152.14 (7)
H54B—C54—H54C	109.5	O16—Mn1—O18	160.49 (8)
C54—O23—H23	109.5	O13—Mn1—O18	88.11 (7)
C26—C25—C24	119.3 (3)	O4—Mn1—O18	87.35 (7)
C26—C25—H25	120.3	O16—Mn1—O1	78.77 (7)
C24—C25—H25	120.3	O13—Mn1—O1	75.90 (7)
C25—C26—C27	120.7 (3)	O4—Mn1—O1	77.41 (7)
C25—C26—H26	119.6	O18—Mn1—O1	97.05 (7)
C27—C26—H26	119.6	O16—Mn1—O7	106.50 (8)
C26—C27—C28	121.3 (3)	O13—Mn1—O7	128.67 (7)
C26—C27—H27	119.4	O4—Mn1—O7	73.93 (7)
C28—C27—H27	119.4	O18—Mn1—O7	67.10 (7)
O12—C28—C23	123.3 (3)	O1—Mn1—O7	147.62 (7)
O12—C28—C27	117.9 (3)	O16—Mn1—O10	82.52 (7)
C23—C28—C27	118.7 (3)	O13—Mn1—O10	76.74 (7)
O14—C29—N5	120.5 (3)	O4—Mn1—O10	130.22 (7)
O14—C29—C30	118.9 (2)	O18—Mn1—O10	111.37 (7)
N5—C29—C30	120.6 (3)	O1—Mn1—O10	139.44 (7)
C31—C30—C35	119.0 (3)	O7—Mn1—O10	72.43 (7)
C31—C30—C29	118.0 (3)	O15—Mn2—O1	173.87 (10)
C35—C30—C29	122.8 (3)	O15—Mn2—O2	96.26 (9)
C32—C31—C30	121.2 (3)	O1—Mn2—O2	81.69 (8)
C32—C31—H31	119.4	O15—Mn2—N5	91.32 (9)
C30—C31—H31	119.4	O1—Mn2—N5	90.85 (9)
C31—C32—C33	119.7 (3)	O2—Mn2—N5	172.40 (9)
C31—C32—H32	120.2	O15—Mn2—N6	92.78 (11)
C33—C32—H32	120.2	O1—Mn2—N6	92.94 (10)
C34—C33—C32	120.3 (3)	O2—Mn2—N6	88.75 (10)
C34—C33—H33	119.8	N5—Mn2—N6	90.18 (10)
C32—C33—H33	119.8	O15—Mn2—O20	85.86 (10)
C33—C34—C35	121.4 (3)	O1—Mn2—O20	88.30 (9)
C33—C34—H34	119.3	O2—Mn2—O20	88.41 (9)
C35—C34—H34	119.3	N5—Mn2—O20	92.85 (10)
O15—C35—C34	117.2 (3)	N6—Mn2—O20	176.70 (10)
O15—C35—C30	124.3 (3)	O3—Mn3—O4	178.26 (10)
C34—C35—C30	118.4 (3)	O3—Mn3—N1	89.50 (10)
C36—N6—C38	107.7 (5)	O4—Mn3—N1	91.88 (9)
C36—N6—Mn2	120.9 (3)	O3—Mn3—N8	89.10 (10)
C38—N6—Mn2	131.3 (5)	O4—Mn3—N8	90.09 (10)
N6—C36—N7	108.9 (7)	N1—Mn3—N8	155.17 (11)
N6—C36—H36	125.5	O3—Mn3—O5	99.25 (10)
N7—C36—H36	125.5	O4—Mn3—O5	79.41 (8)
C36—N7—C37	107.1 (7)	N1—Mn3—O5	102.64 (10)
C36—N7—H7	126.5	N8—Mn3—O5	102.06 (10)
C37—N7—H7	126.5	O6—Mn4—O7	178.56 (9)
C38—C37—N7	107.3 (8)	O6—Mn4—N2	88.36 (10)
C38—C37—H37	126.4	O7—Mn4—N2	93.07 (9)
N7—C37—H37	126.4	O6—Mn4—N10	87.87 (10)
C37—C38—N6	108.8 (10)	O7—Mn4—N10	90.69 (9)
C37—C38—H38	125.6	N2—Mn4—N10	169.38 (10)
N6—C38—H38	125.6	O6—Mn4—O8	103.11 (9)
N7B—C36B—H36B	123.2	O7—Mn4—O8	76.92 (8)
C36B—N7B—C37B	104.0 (15)	N2—Mn4—O8	96.43 (9)
C36B—N7B—H7B	128.0	N10—Mn4—O8	94.10 (9)
C37B—N7B—H7B	128.0	O6—Mn4—O18	112.80 (9)
N7B—C37B—C38B	109.3 (14)	O7—Mn4—O18	67.32 (7)
N7B—C37B—H37B	125.4	N2—Mn4—O18	80.07 (8)
C38B—C37B—H37B	125.4	N10—Mn4—O18	92.27 (8)
C37B—C38B—H38B	125.3	O8—Mn4—O18	143.72 (7)
N8—C39—N9	111.1 (3)	O9—Mn5—O10	176.73 (9)
N8—C39—H39	124.4	O9—Mn5—N3	88.83 (10)
N9—C39—H39	124.4	O10—Mn5—N3	91.64 (9)
C41—C40—N9	106.4 (3)	O9—Mn5—N12	89.89 (10)
C41—C40—H40	126.8	O10—Mn5—N12	89.53 (10)
N9—C40—H40	126.8	N3—Mn5—N12	177.84 (11)
C40—C41—N8	109.4 (3)	O9—Mn5—O11	98.51 (9)
C40—C41—H41	125.3	O10—Mn5—O11	78.26 (8)
N8—C41—H41	125.3	N3—Mn5—O11	89.90 (9)
N11—C42—N10	110.1 (3)	N12—Mn5—O11	88.56 (10)
N11—C42—H42	125.0	O9—Mn5—O17	90.76 (9)
N10—C42—H42	125.0	O10—Mn5—O17	92.45 (8)
N11—C43—C44	107.1 (3)	N3—Mn5—O17	92.74 (9)
N11—C43—H43	126.4	N12—Mn5—O17	89.02 (10)
C44—C43—H43	126.4	O11—Mn5—O17	170.42 (8)
C43—C44—N10	108.2 (3)	O12—Mn6—O13	175.85 (9)
C43—C44—H44	125.9	O12—Mn6—N4	90.68 (9)
N10—C44—H44	125.9	O13—Mn6—N4	93.46 (8)
N12—C45—N13	110.3 (4)	O12—Mn6—O14	93.81 (9)
N12—C45—H45	124.9	O13—Mn6—O14	82.09 (8)
N13—C45—H45	124.9	N4—Mn6—O14	172.99 (9)
C47—C46—N13	105.9 (4)	O12—Mn6—N14	89.54 (10)
C47—C46—H46	127.0	O13—Mn6—N14	89.85 (9)
N13—C46—H46	127.0	N4—Mn6—N14	94.50 (10)
C46—C47—N12	108.9 (4)	O14—Mn6—N14	90.91 (9)
C46—C47—H47	125.5	O12—Mn6—O19	93.23 (9)
N12—C47—H47	125.5	O13—Mn6—O19	87.05 (8)
N14—C48—N15	111.0 (3)	N4—Mn6—O19	90.17 (9)
N14—C48—H48	124.5	O14—Mn6—O19	84.21 (8)
N15—C48—H48	124.5	N14—Mn6—O19	174.54 (9)
			
N1—C1—C2—C3	−179.8 (3)	O6—C14—C9—C10	178.5 (3)
O2—C1—C2—C3	0.5 (4)	C13—C14—C9—C10	−1.1 (4)
N1—C1—C2—C7	−0.8 (5)	O6—C14—C9—C8	−2.2 (5)
O2—C1—C2—C7	179.4 (3)	C13—C14—C9—C8	178.2 (3)
C7—C2—C3—C4	1.8 (5)	O5—C8—C9—C10	−2.0 (4)
C1—C2—C3—C4	−179.2 (3)	N2—C8—C9—C10	176.4 (3)
C2—C3—C4—C5	0.3 (6)	O5—C8—C9—C14	178.7 (3)
C3—C4—C5—C6	−1.8 (6)	N2—C8—C9—C14	−2.9 (4)
C4—C5—C6—C7	1.2 (6)	N11—C42—N10—C44	−0.7 (4)
C5—C6—C7—O3	−178.0 (3)	N11—C42—N10—Mn4	−177.3 (2)
C5—C6—C7—C2	0.9 (5)	C43—C44—N10—C42	−0.1 (4)
C3—C2—C7—O3	176.4 (3)	C43—C44—N10—Mn4	176.4 (2)
C1—C2—C7—O3	−2.5 (5)	N10—C42—N11—C43	1.3 (4)
C3—C2—C7—C6	−2.4 (5)	C44—C43—N11—C42	−1.4 (4)
C1—C2—C7—C6	178.7 (3)	N13—C45—N12—C47	−0.7 (5)
C9—C10—C11—C12	−0.4 (5)	N13—C45—N12—Mn5	−171.7 (3)
C10—C11—C12—C13	−1.0 (5)	C46—C47—N12—C45	0.1 (6)
C11—C12—C13—C14	1.4 (5)	C46—C47—N12—Mn5	171.4 (3)
C12—C13—C14—O6	−179.9 (3)	N12—C45—N13—C46	0.9 (5)
C12—C13—C14—C9	−0.3 (5)	C47—C46—N13—C45	−0.8 (6)
O8—C15—C16—C17	11.9 (4)	N15—C48—N14—C50	−0.3 (4)
N3—C15—C16—C17	−167.5 (3)	N15—C48—N14—Mn6	173.6 (2)
O8—C15—C16—C21	−168.2 (3)	C49—C50—N14—C48	−0.4 (5)
N3—C15—C16—C21	12.4 (4)	C49—C50—N14—Mn6	−173.7 (3)
C21—C16—C17—C18	1.1 (5)	N14—C48—N15—C49	0.8 (4)
C15—C16—C17—C18	−179.0 (3)	C50—C49—N15—C48	−1.0 (5)
C16—C17—C18—C19	1.0 (5)	C1—N1—O1—Mn2	−0.3 (3)
C17—C18—C19—C20	−1.0 (5)	Mn3—N1—O1—Mn2	−169.55 (11)
C18—C19—C20—C21	−1.1 (5)	C1—N1—O1—Mn1	−178.42 (18)
C17—C16—C21—O9	178.0 (3)	Mn3—N1—O1—Mn1	12.4 (3)
C15—C16—C21—O9	−1.9 (5)	N1—C1—O2—Mn2	2.5 (3)
C17—C16—C21—C20	−3.1 (4)	C2—C1—O2—Mn2	−177.8 (2)
C15—C16—C21—C20	176.9 (3)	C6—C7—O3—Mn3	−159.5 (2)
C19—C20—C21—O9	−177.9 (3)	C2—C7—O3—Mn3	21.7 (5)
C19—C20—C21—C16	3.2 (5)	C8—N2—O4—Mn3	−9.4 (3)
O11—C22—C23—C28	166.5 (3)	Mn4—N2—O4—Mn3	173.86 (10)
N4—C22—C23—C28	−14.0 (4)	C8—N2—O4—Mn1	134.77 (19)
O11—C22—C23—C24	−9.8 (4)	Mn4—N2—O4—Mn1	−41.9 (2)
N4—C22—C23—C24	169.7 (3)	N2—C8—O5—Mn3	4.1 (3)
C28—C23—C24—C25	0.4 (5)	C9—C8—O5—Mn3	−177.5 (2)
C22—C23—C24—C25	176.9 (3)	C13—C14—O6—Mn4	−169.7 (2)
C23—C24—C25—C26	−2.0 (6)	C9—C14—O6—Mn4	10.7 (4)
C24—C25—C26—C27	1.7 (6)	C15—N3—O7—Mn4	−2.3 (3)
C25—C26—C27—C28	0.2 (6)	Mn5—N3—O7—Mn4	−177.05 (10)
C24—C23—C28—O12	−179.5 (3)	C15—N3—O7—Mn1	−133.94 (19)
C22—C23—C28—O12	4.4 (5)	Mn5—N3—O7—Mn1	51.33 (19)
C24—C23—C28—C27	1.4 (5)	N3—C15—O8—Mn4	−0.9 (3)
C22—C23—C28—C27	−174.8 (3)	C16—C15—O8—Mn4	179.8 (2)
C26—C27—C28—O12	179.1 (3)	C16—C21—O9—Mn5	−17.3 (4)
C26—C27—C28—C23	−1.7 (5)	C20—C21—O9—Mn5	163.9 (2)
O14—C29—C30—C31	−7.8 (4)	C22—N4—O10—Mn5	−3.3 (3)
N5—C29—C30—C31	172.7 (3)	Mn6—N4—O10—Mn5	179.56 (10)
O14—C29—C30—C35	168.7 (3)	C22—N4—O10—Mn1	124.3 (2)
N5—C29—C30—C35	−10.8 (4)	Mn6—N4—O10—Mn1	−52.82 (17)
C35—C30—C31—C32	0.7 (5)	N4—C22—O11—Mn5	3.6 (3)
C29—C30—C31—C32	177.3 (3)	C23—C22—O11—Mn5	−176.9 (2)
C30—C31—C32—C33	0.0 (5)	C23—C28—O12—Mn6	23.3 (4)
C31—C32—C33—C34	−0.4 (6)	C27—C28—O12—Mn6	−157.5 (2)
C32—C33—C34—C35	0.1 (5)	C29—N5—O13—Mn6	10.7 (3)
C33—C34—C35—O15	−177.2 (3)	Mn2—N5—O13—Mn6	−177.26 (10)
C33—C34—C35—C30	0.6 (5)	C29—N5—O13—Mn1	144.84 (19)
C31—C30—C35—O15	176.6 (3)	Mn2—N5—O13—Mn1	−43.1 (2)
C29—C30—C35—O15	0.2 (5)	N5—C29—O14—Mn6	−2.9 (3)
C31—C30—C35—C34	−1.0 (4)	C30—C29—O14—Mn6	177.6 (2)
C29—C30—C35—C34	−177.4 (3)	C34—C35—O15—Mn2	−164.4 (2)
C38—N6—C36—N7	2.9 (19)	C30—C35—O15—Mn2	18.0 (4)
Mn2—N6—C36—N7	−175.3 (5)	O17—C79—O16—Mn1	−10.1 (6)
N6—C36—N7—C37	−0.5 (11)	O16—C79—O17—Mn5	−3.2 (5)
C36—N7—C37—C38	−2.2 (18)	O19—C77—O18—Mn1	7.2 (5)
N7—C37—C38—N6	4 (3)	O19—C77—O18—Mn4	123.5 (3)
C36—N6—C38—C37	−4 (3)	O18—C77—O19—Mn6	−1.8 (5)
Mn2—N6—C38—C37	173.7 (9)	C35—O15—Mn2—O2	161.5 (3)
C36B—N7B—C37B—C38B	1 (4)	C35—O15—Mn2—N5	−19.2 (3)
N9—C40—C41—N8	−0.5 (6)	C35—O15—Mn2—N6	−109.5 (3)
N11—C43—C44—N10	0.9 (4)	C35—O15—Mn2—O20	73.5 (3)
N13—C46—C47—N12	0.4 (6)	N1—O1—Mn2—O2	1.25 (17)
N15—C49—C50—N14	0.9 (5)	Mn1—O1—Mn2—O2	179.29 (13)
O2—C1—N1—O1	−1.5 (4)	N1—O1—Mn2—N5	−177.28 (18)
C2—C1—N1—O1	178.8 (2)	Mn1—O1—Mn2—N5	0.76 (13)
O2—C1—N1—Mn3	166.4 (2)	N1—O1—Mn2—N6	−87.06 (18)
C2—C1—N1—Mn3	−13.3 (4)	Mn1—O1—Mn2—N6	90.98 (13)
O5—C8—N2—O4	2.9 (4)	N1—O1—Mn2—O20	89.89 (17)
C9—C8—N2—O4	−175.4 (2)	Mn1—O1—Mn2—O20	−92.07 (13)
O5—C8—N2—Mn4	178.8 (2)	C7—O3—Mn3—N1	−26.1 (3)
C9—C8—N2—Mn4	0.5 (4)	C7—O3—Mn3—N8	178.7 (3)
O8—C15—N3—O7	2.0 (4)	C7—O3—Mn3—O5	76.6 (3)
C16—C15—N3—O7	−178.6 (2)	N2—O4—Mn3—N1	111.33 (18)
O8—C15—N3—Mn5	175.40 (19)	Mn1—O4—Mn3—N1	−30.76 (12)
C16—C15—N3—Mn5	−5.2 (4)	N2—O4—Mn3—N8	−93.42 (18)
O11—C22—N4—O10	−0.5 (4)	Mn1—O4—Mn3—N8	124.48 (12)
C23—C22—N4—O10	180.0 (2)	N2—O4—Mn3—O5	8.83 (17)
O11—C22—N4—Mn6	176.1 (2)	Mn1—O4—Mn3—O5	−133.26 (12)
C23—C22—N4—Mn6	−3.4 (4)	C14—O6—Mn4—N2	−10.1 (3)
O14—C29—N5—O13	−5.1 (4)	C14—O6—Mn4—N10	160.0 (3)
C30—C29—N5—O13	174.4 (2)	C14—O6—Mn4—O8	−106.3 (3)
O14—C29—N5—Mn2	−175.84 (19)	C14—O6—Mn4—O18	68.5 (3)
C30—C29—N5—Mn2	3.7 (4)	C21—O9—Mn5—N3	18.6 (3)
N9—C39—N8—C41	0.0 (4)	C21—O9—Mn5—N12	−163.1 (3)
N9—C39—N8—Mn3	180.0 (2)	C21—O9—Mn5—O11	108.3 (3)
C40—C41—N8—C39	0.3 (5)	C21—O9—Mn5—O17	−74.1 (3)
C40—C41—N8—Mn3	−179.7 (3)	C28—O12—Mn6—N4	−30.0 (3)
N8—C39—N9—C40	−0.3 (5)	C28—O12—Mn6—O14	144.6 (2)
C41—C40—N9—C39	0.5 (5)	C28—O12—Mn6—N14	−124.5 (3)
C11—C10—C9—C14	1.5 (5)	C28—O12—Mn6—O19	60.2 (2)
C11—C10—C9—C8	−177.9 (3)		

### Hydrogen-bond geometry (Å, º)

**Table:**

*D*—H···*A*	*D*—H	H···*A*	*D*···*A*	*D*—H···*A*
C44—H44···O11	0.95	2.47	3.413 (4)	173
C47—H47···N14	0.95	2.68	3.599 (5)	162
C51—H51*A*···O18	0.98	2.50	3.360 (4)	147
N7—H7···O25*B*	0.88	1.88	2.658 (9)	147
N9—H9···O17^i^	0.88	2.07	2.898 (3)	156
N11—H11*A*···O14^ii^	0.88	2.00	2.869 (3)	168
N13—H13*A*···O2^iii^	0.88	1.99	2.827 (4)	159
N15—H15···O8^iv^	0.88	1.96	2.800 (4)	159
N7*B*—H7*B*···O21	0.88	2.11	2.933 (15)	155
O20—H20*A*···O22^v^	0.85 (2)	1.85 (2)	2.681 (5)	168 (5)
O20—H20*A*···O22*B*^v^	0.85 (2)	1.96 (4)	2.75 (3)	155 (4)
O22—H22*A*···O24	0.84	1.97	2.746 (7)	154
O24—H24*A*···O21	0.84	2.03	2.808 (6)	154
O25*B*—H25*A*···O23*B*	0.84	1.91	2.601 (9)	138
O22*B*—H22*C*···O21*B*^vi^	0.84	2.48	3.29 (4)	160
O23—H23···O12^vii^	0.84	2.19	2.828 (9)	133
O23*B*—H23*B*···O12^vii^	0.84	2.08	2.897 (6)	165

Symmetry codes: (i) −*x*+1, −*y*+1, −*z*+1; (ii) −*x*+1, −*y*+1, −*z*; (iii) *x*−1, *y*, *z*; (iv) −*x*+1, *y*+1/2, −*z*+1/2; (v) *x*, −*y*+3/2, *z*−1/2; (vi) −*x*+2, −*y*+2, −*z*+1; (vii) *x*, −*y*+3/2, *z*+1/2.
